# The New York Street Cleaning Bill

**Published:** 1881-07

**Authors:** 


The Street Cleaning Bill, recently passed by the New York Legislature, if properly carried into operation, cannot fail to confer hygienic advantages upon the metropolis, of the greatest importance.

